# Who Likes Unhealthy Food with a Strong Flavour? Influence of Sex, Age, Body Mass Index, Smoking and Olfactory Efficiency on Junk Food Preferences

**DOI:** 10.3390/nu14194098

**Published:** 2022-10-02

**Authors:** Magdalena Hartman-Petrycka, Joanna Witkoś, Agata Lebiedowska, Barbara Błońska-Fajfrowska

**Affiliations:** 1 Department of Basic Biomedical Science, Faculty of Pharmaceutical Sciences in Sosnowiec, Medical University of Silesia, 41-200 Sosnowiec, Poland; 2 Faculty of Medicine and Health Science, Andrzej Frycz Modrzewski Krakow University, 30-705 Kraków, Poland

**Keywords:** food preferences, junk food, ultra-processed food, fast food, sugary carbonated drinks, sex, age, body mass index, smoking and smell

## Abstract

Background. Unhealthy food is an important element in the development of diseases of civilisation. The aim of this study was to determine how sex, age, body mass index, smoking and olfactory efficiency influence the consumption of such foods. Methods. A total of 283 people living in the Silesian Voivodeship in Poland took part in the study. They were aged 18–82. An interview and olfactory tests were conducted together with assessments of food preferences from 25 types of food products. The extent to which sex, age, body mass index, tobacco addiction and sense of smell influence unhealthy food consumption was assessed. Results. Using the VARIMAX factor analysis, a coherent group of ‘unhealthy food with a strong flavour’ products was selected: crisps, salty snacks, fast food, sugary carbonated drinks and sour products. Unhealthy food was liked more by people who were younger (B = −0.04; PU = −0.05, −0.03; t = −7.43, eta2 = 0.17; *p* < 0.001) and who had a higher BMI (B = 0.03; PU ≤ 0.01, 0.06; t = 1.92, eta2 = 0.01; *p* = 0.056). The efficiency of the sense of smell does not play a significant role in the preference for the ‘unhealthy food’ group as a whole. The analysis of each type of unhealthy food separately showed that young people liked crisps, salty snacks, fast food and sugary carbonated drinks more, men liked fast food and sugary carbonated drinks more than women, and people with a good sense of smell liked sour products. Conclusion. According to the food preferences stated, dietary education should be targeted at young people, especially young men, to prevent the development of overweight and obesity.

## 1. Introduction

Globalisation and economic development have contributed to the modification of both the nutritional profile and eating habits of entire human populations. Since the 20th century, traditional fresh and home-prepared food comprising minimally processed products rich in essential nutrients has largely been replaced by pre-prepared, widely available and affordable ultra-processed food—UPF, also referred to as ‘junk food’ [1,2,3]. The simple methods of processing food and extending its shelf life practised at home have been known to mankind for a long time, but, over the past 100 years, food processing has become industrialised. UPF requires minimal preparation time before consumption. Being both attractive and convenient, it is consequently popular with a significant proportion of the population worldwide [4,5]. Between 1990 and 2010, UPF consumption increased from 11% to 32% of daily energy intake [6]. 

The definition of UPF includes food products and beverages made from food ingredients, often extracted, which are successively modified through a series of mechanochemical processes, such as grinding, heating, freezing, centrifuging, frying, drying, thickening, compressing, irradiating and microwaving, after which the ingredients are mixed and combined into a ready-to-eat food [7]. UPF contains virtually no natural, intact food sources. UPF foods include hydrogenated oils and fats, modified starches, hydrolysed proteins and ground, or extruded ‘blends’ of offal, or meat scraps, and also contain flavourings, colourings and other additives to give it ‘hyper-flavour’ [8,9]. In some cases, UPF is further ‘enriched’ with those dietary components that were initially lost during its production, but, in many cases, the added nutrients exceed the amount found in the natural food. Simple examples of food enrichment that takes place during food production are, for example, the addition of B vitamins, including folic acid, to bread, or vitamins A and D to milk [10].

The wide variety of industrially processed food products include those bought in supermarkets and fast food outlets. Examples of UPF include, but are not limited to: reconstituted meat products (cold cuts, sausages, hamburgers), industrialised packaged bread (e.g., pre-packaged, sliced and plastic-wrapped), pre-prepared ready meals, frozen meals (e.g., pizza), sweetened soft drinks, breakfast cereals, confectionery (cakes, biscuits, buns), salty snacks (e.g., crisps, crackers) and chips (fries), hot dogs and kebabs, as well as artificially combined, very often low-quality, cuts of poultry or fish [8,11]. UPF is often pre-packaged and therefore considered microbiologically safe. Furthermore, due to the ‘enhancers’ added during production, it has a long shelf life and is usually very tasty [8]. It contains significantly more sugar, salt, saturated fats, trans fats and refined grains than unprocessed or minimally processed foods, and is deficient in nutrients, vitamins, fibre and micronutrients [12,13]. UPF is believed to contribute to a high energy intake, at 30% and 50% in middle- and high-income countries respectively. However, it does have an impact on the safeguarding of so-called food security, i.e., securing the availability of sufficient food for the majority of people in developed and developing countries [14]. 

The purchase and consumption of food and drinks outside the home has become a popular and alternative way to eat. Studies have shown that 2.5 billion people worldwide consume so-called ‘street food’ [15]. Popular ‘fast food’ (FF) is defined as food purchased in a self-service restaurant or in eating establishments without a waiting service. Certainly, the widespread availability and abundance of FF restaurants has contributed to the increased consumption of this type of food in most societies around the world. However, consuming this type of food significantly reduces the nutritional quality of the daily diet and is associated with a high calorie intake [16,17]. These foods are usually based on large amounts of red, processed meat, potato chips (fries) and sugar-sweetened drinks. Typically, such dishes are also served as large (extra large) set meals, which further increases the calorie intake of the potential FF restaurant customer [18].

An excess of unhealthy food sources and an increase in the calorie content of the diet, as well as limited access to healthier alternative food options plus accompanying unhealthy eating behaviours, such as eating out, snacking, or even overeating contribute to a number of problems related to the health of human populations [15,16]. 

UPF is now the dominant component in the diet of people worldwide [12]. Studies [19] conducted in ten European countries found that UPF contributes 61–79% to the average energy intake. In the USA, Canada, New Zealand and Latin American countries, such products account for between 25% and 60% of the average daily energy intake [20]. More detailed data are presented in Bains et al. [21] which indicate that the percentage of energy intake from UPF among adults is 45.1–51.9% in Canada, 55.5–56.1% in the USA, 53–54.3% in the UK and 29.9–35.9% in France. Lower proportions of UPF in the diet were reported in Spain 24.4%, Lebanon 27.1%, Brazil 20–29.6% and Malaysia 23%. 

Other data [22] from the US National Health and Nutrition Examination Survey showed that between 2013 and 2016, the percentage of adults consuming fast food on a single day was 36.6%, and between 2015 and 2018, the percentage of children and adolescents aged 2–19 years consuming FF foods on a given day was 36.3%, with adolescents aged 12–19 years consuming a higher percentage of calories from FF than children aged 2–11 years [23]. 

Rising rates of overweight and obesity worldwide, coupled with an increase in diseases related to human eating habits, have directed scientific attention to the quality of products supplied by the food industry [24]. Monteiro et al. [8], from the University of São Paulo, developed the NOVA scale, which allows a society’s health status to be assessed according to the level of food processing present in a country. It separates food and beverages into four groups according to the extent and purpose of the industrial processing. The fourth group consists of UPF, which contains almost no intact food [7,25]. 

FF foods have a high glycaemic index, therefore it has been suggested that this type of food may affect satiety control and glycaemic response which may also provide a higher amount of energy than the human body’s daily requirement [26]. Studies [12] have shown that the average energy density of a meal at a FF restaurant is approximately more than double the energy density of a healthy meal. Reduced physical activity and increased UPF intake has been referred to as the ‘big two’ and is widely regarded as a cause of the obesity epidemic [27].

An estimated 2.8 million people die each year from obesity-related causes [28]. Research by Juul et al. [29] assessing the proportion of UPF-derived calories in relation to obesity found that the prevalence of overweight increased in men from 35% to 56% and in women from 26% to 39%. Obesity increased in men from 4.5% to 11% and in women from 5% to 10%, which can lead to further health consequences, such as cardiovascular disease, stroke, diabetes, hypertension, dyslipidaemia, respiratory disorders and certain types of cancer [28].

Taking these facts into account, it was decided to analyse which factors may contribute to a higher consumption of ‘unhealthy food with a strong flavour’ and to identify the target groups of people to whom prevention programmes should be directed. 

## 2. Materials and Methods

The study involved 283 people aged between 18 and 82, living in the Silesian Voivodeship, mainly in the Górnośląsko-Zagłębiowska Metropolis in Poland. The majority of young people participating in the study were recruited among students of medical analysis, pharmacy, cosmetology and biotechnology of the Faculty of Pharmaceutical Sciences of the Medical University of Silesia. As the research was conducted within the Students’ Science Club, a significant part of the participants were the students’ family and friends. There were 190 women and 93 men, including 43 smokers, who participated in the study. The percentage of people belonging to each BMI category, according to WHO [30], was as follows: 7.8% underweight (<18.5 kg/m^2^), 66.0% normal weight (18.5–24.9 kg/m^2^), 17.0% overweight (25.0–29.9 kg/m^2^), 7.8% first degree obesity (30.0–34.9 kg/m^2^), 1.4% second degree obesity (35.0–39.9 kg/m^2^). Full characteristics of the respondents are summarized in Table 1.

In accordance with the Declaration of Helsinki, all subjects were informed about the purpose and method of the study and gave written consent to participate. The Bioethics Committee of the Silesian Medical University agreed to conduct the study (Resolution KNW/0022/KB1/47/12).

Exclusion criteria for the study were lack of nasal patency as demonstrated by a rhinomanometric test (total flow through the anterior nostrils less than 280 cm^3^), inability to understand the procedures during the study, and refusal to participate in the study. The exclusion of subjects from the study who did not have nasal patency was intended to eliminate such people in whom obstruction could cause short-term olfactory impairment. Pregnant women did not participate in the study.

Preparation of the volunteers for participation in the olfactory tests: Each participant was asked to avoid foods and spices with a strong taste and smell, e.g., garlic, the day before the test, to take special care with their personal hygiene and not to use cosmetics with a strong smell. The study was conducted after a 30-min acclimatisation to the olfactometric laboratory conditions. During this time, no food or drink (other than still water), chewing gum, smoking, applying cosmetics, or engaging in physical exertion was allowed. 

Determination of the olfactory sensitivity threshold: The olfactory sensitivity threshold to n-butanol was assessed by an ECOMA T08 olfactometer using the dynamic olfactometry method in accordance with PN-EN 13725:2007. The olfactometer diluted n-butanol at a concentration of 59.9 ppm with air and administered it to the participants’ stations in the following dilution steps 2^16^, 2^15^,..., 2^3^, 2^2^. Odour samples alternated with air were sent to the test subjects’ stations at a speed of 0.2 m/s, for 2.2 s. The participants’ task was to press a button when they smelled an odour other than air. The measuring cycle was stopped when the odour substance was correctly indicated at least twice and no error was recorded if the air sample was selected. Before the test, the participants did not know the type of substance used. The assessment of the olfactory sensitivity threshold was carried out twice, the lower of the sensitivity thresholds obtained was chosen as the final result.

Identification test of smell: After a 15-min break, the participants moved to a separate room where they were asked to judge which odour they could smell, based on the smell of a substance which had been applied to a smelling strip. Limonene, which smells like citrus, menthol, which smells like mint, phenethyl alcohol, which smells like flowers, eugenol, which smells like cloves, and n-butanol, which smells like an alcoholic chemical, were used in the identification test. The respondents described the names of the odours with no further prompting; names similar to those presented above were accepted, e.g., limonene—lemon, lime, orange, citrus, lemonade. The outcome of the trial was the number of correctly recognised odours.

Food preference test: The food preference test was conducted prior to the olfactory test during the acclimatisation to the olfactometric laboratory conditions. The volunteers viewed a photo album with pictures of twenty-four types of food and sugary carbonated drinks. They were asked to state how pleasant they found the food they were looking at. They marked their answer on 10 cm linear scales labelled at one end ‘0—not at all pleasant’, and at the other end ‘10—maximally pleasant’. The score was the distance from zero to the point marked by the subject. The types of food assessed were: fish dishes, egg dishes, sweet desserts, chocolate, sweets and jellybeans, crisps, dumplings, pasta, milk soup (this is a sweet dish made by pouring hot milk over things such as: boiled rice, pasta, oatmeal, chocolate chips or corn flakes etc.), milk drinks, cheese, vegetables and salads, fruit, sausages and ham, beef and pork, poultry, bread, fast food, salty products, sour products, broth, soups, spicy dishes, seafood and sugary carbonated drinks.

Statistical Analysis: Statistical analysis was carried out using SPSS 21 software. A descriptive analysis was performed, then KMO (Kaiser-Meyer-Olkin) indices were checked for all the tested food, Bartlett’s test was performed and a factor analysis of the main components was carried out using VARIMAX rotation. After seven factors were identified, factor one was named ‘unhealthy food with a strong flavour’ for the purposes of further analysis in the research. Regression models were built for the entire ‘unhealthy food with a strong flavour’ group and for each component independently, i.e., crisps, salty products, fast food, sour products, sugary carbonated drinks. The predictors in the regression models were sex, age, BMI, pack-year, olfactory sensitivity and odour identification. Non-standardised regression coefficients (B) were given for numerical and dichotomous predictors, along with 95% confidence intervals. A coefficient of multiple determination (R2) value was also provided for each analysis, in addition to effect-size ratios for each predictor; these were expressed as eta-square. In the case of smoking, the indicator of the ‘pack-years’ addiction (number of cigarette packs smoked per day times years of smoking) was used as a predictor. 

## 3. Results

**Figure 1:**
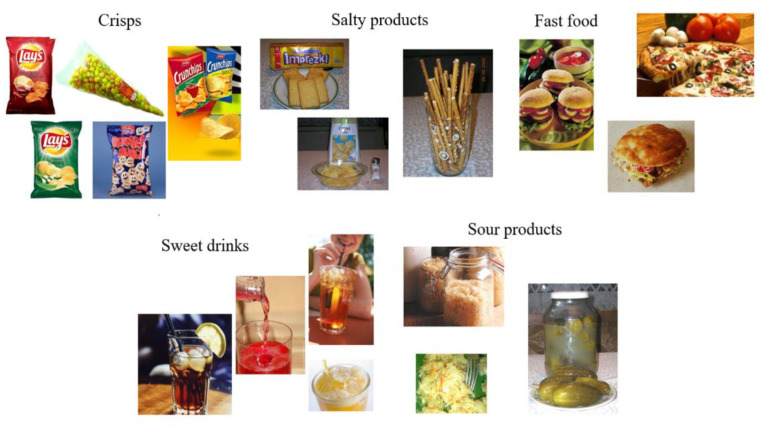
Foods which comprised Factor One—‘unhealthy food with a strong flavour’.

A total of 25 types of food were analysed in order to assess the preferences, so a factor analysis was performed using the principal component method with VARIMAX rotation. This technique allows a larger number of variables to be categorised into certain groups, such that the variables within each group relate to a similar factor. The KMO value was 0.80, so it was acceptable [31]. Thanks to Bartlett’s test of sphericity, the hypothesis that the individual items are uncorrelated and that there was no factor structure among them (chi2 = 2408.64, df = 300, *p* < 0.001) was rejected. Only factors for which the eigenvalue exceeded one were included. A clear factor solution was obtained. Overall, the seven factors identified explained a total of 62.10% of the variance for the scale items. Factor one had the highest eigenvalue of 2.92 and explained 11.66 % of the variance value. The factor loadings after VARIMAX rotation for factor one, named in this study as ‘unhealthy food with a strong flavour’, were crisps with a value of 0.76, salty snacks 0.76, fast food 0.75, sugary carbonated drinks 0.61 and sour products 0.40. The foods that comprised factor one ‘unhealthy food with a strong flavour’ are shown in Figure 1 and the values of the declared pleasure of eating these foods are presented in Table 2.

Factor One ‘unhealthy food with a strong flavour’ became the dependent variable in the regression model (Table 3). The value of the multiple coefficient of determination in this model is R2c = 0.20. Age had the greatest impact on the increased preference for unhealthy food with strong flavour; the younger the subjects were, the greater the declared derived pleasure from unhealthy food with a strong flavour (B = −0.04; PU = −0.05, −0.03; t = −7.43, eta2 = 0.17; *p* < 0.001). Some effect of BMI on the preference for unhealthy food with a strong flavour was also observed, such products being more liked by those with a higher BMI (B = 0.03; PU ≤ 0.01, 0.06; t = 1.92, eta2 = 0.01; *p* = 0.056) and with a stronger tobacco habit expressed in pack-years (B = 0.02; PU ≤ 0.01, 0.03; t = 1.77, eta2 = 0.01; *p* = 0.078).

From the analysis of the influence of factors on the particular type of food categorised as ‘unhealthy food with a strong flavour’, age was seen as a significant effect (Table 4). The younger the subjects were, the more pleasure they declared from eating crisps (B = −0.12; PU = −0.15, −0.09; t = −7.45, eta2 = 0.17; *p* < 0.001), salty snacks (B = −0.07; PU = −0. 10, −0.04; t = −4.11, eta2 = 0.06; *p* < 0.001), fast food (B = −0.14; PU = −0.17, −0.11; t = −8.22, eta2 = 0.20; *p* < 0.001) and sugary carbonated drinks (B = −0.06; PU = −0.09, −0.02; t = −3.38, eta2 = 0.04; *p* = 0.001). In addition, it was observed that the sex of the participant had an effect on fast food and sugary carbonated drink preferences. The men liked fast food (B = 0.97; PU = 0.18, 1.75; t = 2.43, eta2 = 0.02; *p* = 0.016) and sugary carbonated drinks (B = 1.25; PU = 0.45, 2.05; t = 3.07, eta2 = 0.03; *p* = 0.002) more than the women. The consumption of sour products was significantly influenced by olfactory sensitivity. Those with greater olfactory sensitivity liked sour products more (B ≤ 0.01; PU ≤ 0.01, <0.01; t = 2.29, eta2 = 0.02; *p* = 0.023).

## 4. Discussion

### 4.1. Food Groupings

In this study, food preferences were assessed for 25 types of food and data were collected on sex, age, weight, height and smoking habits. Olfactory performance was assessed using two types of test. The statistical analysis, more specifically VARIMAX factor analysis, showed that the formation of preferences for certain types of food converged and specific groups of foods could be identified. The most convergent results were found in the preferences for foods such as crisps, salty snacks, fast food, sugary carbonated drinks and sour products. What these foods have in common is a high degree of processing, a distinct taste, a high calorie content and a low nutritional value, i.e., characteristics that we attribute to junk food/highly processed food [7,8,9], hence the term ‘unhealthy food with a strong flavour’ used in the results as a name for the entire group. 

Although the formation of a common group comprising salty snacks, crisps and fast food is not surprising, sugary drinks, for taste reasons, fit more closely into the common group formed by chocolate, desserts and sweets. It appears, however, that the preference for sugary drinks is more closely aligned with fast food, crisps and salty snacks rather than other ‘sweets’. This observation is supported by literature. Lian Li et al. and Bains at al. [21,32] report that consumption of FF-type foods, tends to be associated with an increased consumption of carbonated soft drinks, usually sweet-tasting, and a lower consumption of fruit and vegetables. Soft drinks are of concern because they are a major source of free sugar. Over a period of 40 years (1980–2020), the share of total sugar intake from soft drinks increased from 15% to 37%, and further increases in their consumption are continually being reported [21,32]. A publication by Monteiro [8] also cited sugary beverages as an example of highly processed junk food alongside crisps, salty crackers and fast foods.

Sour foods such as pickled gherkins and sauerkraut are very different from other foods in the unhealthy food with a strong flavor group, they are not high in calories, fats or simple sugars, and are processed using natural processes; in addition, their distinct sour taste is due to the natural pickling process and not to spices or flavour enhancers. Due to their vegetable origin, it would seem that the preference for gherkins and sauerkraut should correspond to the preference for fruit and vegetables. The statistical analysis, however, assigned them to Factor One ‘unhealthy food with a strong flavour’, although the factor load was only 0.40. Such an assignment may be due, on the one hand, to their distinct taste, and, on the other hand, to the fact that, in Poland, gherkins are often added to the very popular hamburger. In contrast to crisps, salty snacks and sugary drinks, gherkins and sauerkraut are healthy, due, among other things, to their high vitamin content and beneficial effects on intestinal flora [33]. It should be remembered, however, that a few slices of gherkin in a hamburger are not enough to achieve tangible health benefits. 

### 4.2. Factors Influencing the Preference of the Entire Group of Foods Referred to as Factor One ‘Unhealthy Food with a Strong Flavour’

The regression model showed that young people liked foods from the created ‘unhealthy food with a strong flavour’ group more than those from the older age groups. The reasons for this are complex. In part, it may be culturally conditioned, as junk food only appeared in Poland after the political transformation in the 1990s, before which the older generation of Poles mostly prepared meals from scratch at home on their own. A study by Gramza-Michałowska [34] assessed the relationship between demographic characteristics such as age, BMI, sleep duration, daily activity and dietary preferences, as well as health awareness regarding the consumption of fast food. Age and gender differences were found to be closely associated with FF consumption. There was a tendency for FF food consumption to decrease with age and this was probably related to the level of health awareness of these respondents. It was also noted that people for whom eating lunch was an important part of the day were more likely to enjoy FF foods and this was also replicated at the weekend. The authors concluded that the actual preferences for choosing FF was a young age and a love of eating lunch and this overrode an individual’s lifestyle or body weight. Another reason linked to eating habits is the cumulative exposure to television, which influences viewers’ opinions and beliefs, as well as food choices, especially among young people [35]. Advertisements for fast food, including hamburgers, fries (chips), fried chicken, pizza and sugary carbonated drinks are very common in television programmes. In a study by Powell et al. [36], it was shown that almost 90% of food advertisements seen by teenagers are for products high in fat, sugar, or salt, and 23% of food advertisements refer to FF restaurants. The authors found that advertisements for restaurants serving FF food were the most common advertising product shown during children’s television programmes in as many as 12 countries. The images shown are usually of happy, healthy and smiling families and unrealistically slim people eating high-fat and calorie-dense food, which is an obvious contradiction, but, to a young person, this fact may not be noticeable. In a study by Gearhardt et al. [37], it was shown that food advertisements can influence human behaviour by engaging neurobiological systems associated with reward and thus unconscious, physiological and psychological responses involving specific behaviours, including those of eating. Advertising is not the only factor increasing young people’s consumption of unhealthy foods. Consumers’ choices in relation to the food they buy depend on a number of factors, such as its cost and availability in shops, individual taste, and the knowledge, or lack thereof, of healthy products. A study by Djupegot et al. [9] showed that UPFs are often purchased by people who face time scarcity during the day, as foods for quick preparation are convenient for them and give them a sense of better time management during the day. The above was also confirmed by a study by Sogari et al. [38], which found that the most commonly reported barriers to a healthy diet were time scarcity, the high price of foods, problems with availability and a general lack of motivation to prepare healthy foods. Preparing healthy food was often perceived by respondents as a time-consuming activity, and thus UPF was used as a time-saving strategy. It was chosen both for lunch and as snacks and soft drinks [9,39]. It should also be noted that working hours are often extended these days, which also automatically changes the circadian rhythm of the human body and the time of eating meals. Unfortunately, this convenient UPF consumed in the evening and at night has a particularly detrimental effect on human health, as the metabolism slows down at the end of the day. Eating beyond the 12-h daily rhythm not only disrupts the circadian rhythm, but also increases the risk of metabolic diseases [40]. It can be assumed that older people who have completed their working lives have more time to take care of their diet and age-related illnesses are a motivation to enhance their knowledge and spend time preparing healthier meals.

There was a weak positive relationship between BMI and liking unhealthy food with strong flavor. Each increase in BMI value by 1 [kg/m^2^] increased the declared pleasure of eating by 0.3. As a result, the change in the BMI in the studied group from the mean value 23.33 to 25 (overweight, according to BMI) and 30 (obesity, according to BMI), respectively, translates into an increase in the declared pleasure of eating foods from the Factor One Unhealthy Food group by 0.5 and 2.0, respectively. There have been and also very weak positive association between severity of tobacco addiction expressed in pack-years and liking unhealthy food with strong flavor. Literature provides ample evidence of associations between BMI, tobacco addiction and unhealthy eating. According to Chao et al. [41], cigarette smoking is strongly associated with obesity-related behaviours, including unhealthy diet, thus contributing to a large number of diseases that can reduce life expectancy, such as the aforementioned obesity, especially abdominal obesity, cardiovascular disease, type 2 diabetes, metabolic syndrome and cancer. Unhealthy behaviours associated with smoking include a greater need to eat foods, especially those rich in fat, and a habitual consumption of such foods [41]. Similarly, Palaniappan et al. [42] found an association between cigarette smoking, increased saturated fat intake and increased calorie intake in their study. The reasons for this link between unhealthy eating and cigarette smoking are not fully understood. One reason may be a reduced perception of fat taste, which may affect the taste perception of foods rich in fats [43]. 

An important element of this research was the evaluation of the relationship between the consumption of a whole group of ‘ unhealthy food with a strong flavour ‘ dishes and olfactory performance. No such relationship was found. A detailed analysis of each of the ‘unhealthy foods’ only showed an increased preference for sour foods such as pickled gherkins and sauerkraut in people with a better olfactory sensitivity threshold. At the same time, no such relationship was confirmed in the identification test of smell. In addition, gherkins, as highlighted in the earlier part of the discussion, were included in ‘unhealthy food with a strong flavour’ mainly for statistical reasons, not for being highly processed or high in fats, sugars or calories. Therefore, no links can be demonstrated between olfactory performance and a preference for junk food.

### 4.3. Factors Which Influence the Preferences of Individual Dishes Included in the ‘Unhealthy Food with a Strong Flavour’ Group

Statistical analysis showed that crisps are liked primarily by younger people and this age dependence is very strong, explaining as much as 17% of the variation for the liking of crisps. As with crisps, age plays a strong role in the fondness for salty snacks, explaining the 6% variation in the liking of salty snacks. Young people like salty snacks more than older people. The regression model indicates that a young age explains as much as 20% of the variability in liking fast food. This coincides with the relationships between age and the preference for crisps and salty snacks described above. However, additional correlations can still be observed. A higher rate of fondness for fast food was found in men than in women. Carbonated drinks were mainly liked by younger people with males also playing a significant role, as in the case of fast food. The above data indicate that not only age but also sex is an important factor in the preference for fast food and carbonated drinks. Women are less fond of fast food and sugary drinks. These findings are supported by other studies [44,45] which showed an increased consumption of FF, processed meat and sugary soft drinks by men compared to women. A study by Forsyth et al. [46] found that there are gender differences in dietary choices and consumption. It was shown that young men living near a large number of FF restaurants were more likely to eat at these places than women in the same residential area. Potential reasons for the observed differences were due to young women’s greater concern for their body appearance, including controlling their weight, concerns about possible changes to their appearance and their involvement in preparing healthy meals at home. It has also been noted that FF marketing more strongly targets men [47]. In a study by Anderson et al. [48], it was estimated that 80% of Michigan adults aged 18 to 64 years ate at FF restaurants at least once a month, and 28% consumed such foods regularly. Young adults and men were found to have eaten this type of food most often, and speed and convenience were cited as reasons, with 64% responding in this way. 

The preference for sour foods such as pickled gherkins and sauerkraut was particularly distinct; the degree of fondness for such foods depended on olfactory performance. People with high olfactory sensitivity liked such foods more. This relationship may be related to the fact that pickled foods have a very intense smell, and that the whole bouquet of sensory impressions is important in the perception of food [49].

### 4.4. Limitations

The research was based on the results of a relatively large group of people, but not representative of Poland, as described in the methodology. Due to the participation of a large group of students of medical analytics, pharmacy, cosmetology and biotechnology, compared to the population of Poland, the study group was younger, included a larger number of women, has a lower rate of overweight and obesity, and was characterized by healthier food preferences. For the above reasons, the main goal of the study was not to determine food preferences and indicate the most liked products, although such a list is presented in Table 2, but to find the relationship between the individual characteristics of the respondents and their food preferences. Despite the presented limitations, the applied statistical analysis allows, in the above-mentioned study group, to identify features influencing specific food preferences. 

Another limitation of the study may be that the food preferences were investigated based on participants’ responses regarding their declared pleasure of eating each food, rather than on the basis of an ongoing diet diary from which the real consumption of junk foods could be assessed. Declared food preferences may be loaded with ‘social approval’, with respondents trying to answer according to social expectations in order to better present themselves to the researcher. An example of the influence of social expectations on declared food preferences is presented in a study by Miller et al. [50] in which differences in results were observed depending on the presentation of the purpose of the study. Women who were informed that they were being surveyed about their consumption of healthy fruit and vegetables declared that they ate more of these than when compared to a group in which the research objective was not presented in such detail. In order to minimise the effect of social expectations, the authors of the study emphasised that, in the preference survey, participants were asked to indicate how much they liked a particular food and not how often they ate it. In this way the effect of social expectations was minimised, because it is possible to like eating a particular food very much without necessarily eating it on a regular basis. In addition, the answer sheets were filled in by the respondents themselves to reduce discomfort; it being easier for an overweight and obese person to indicate that he or she likes fast food on a scale of 10 rather than to say so directly in the presence of a researcher. Bearing in mind these limitations, the following summary of the study is presented. 

## 5. Conclusions

People who like crisps also tend to like salty snacks, fast food, sugary carbonated drinks and pickled gherkins, which have been combined into a common group—unhealthy food with a strong flavour. People who like ‘unhealthy food with a strong flavour’ are predominantly those who are young, have a higher BMI and, to some extent, have a higher severity of tobacco addiction. Olfactory performance does not play a significant role in the preference for the unhealthy food with a strong flavour group as a whole. The analysis of individual dishes showed that, in addition to young people, fast food and sugary carbonated drinks were liked more by men than by women and pickled gherkins were liked more by people with an efficient sense of smell.

In order to take care of the health of the population, changes in the food industry should be pursued to improve the quality of the products sold, and preventive programmes on healthy eating should be conducted. According to studies [3,51], better educated people make better nutritional decisions. These programmes should target young people, especially young men. There is a risk that the health status of today’s 20-year-olds in Poland will be worse in 30 years than today’s 50-year-olds, due to dietary misconceptions. 

## Figures and Tables

**Table 1:** Characteristics of the study participants.

	**N**	**Mean**	**Median**	**SD**	**Minimum**	**Maximum**
Age (Years)	283	29.22	23.00	13.44	18.00	82.00
BMI	282	23.33	22.21	4.17	16.65	36.73
No. of years as a smoker	283	2.80	0.00	7.27	0.00	44.00
No. of cigarettes a day	283	2.27	0.00	5.91	0.00	50.00
Severity of addiction (pack-years)	283	1.83	0.00	6.86	0.00	60.00
Olfactory sensitivity threshold (serial dilution)	283	7021	1024	15,208	0.00	65,636
Identification of smell	283	3.94	4.00	1.08	0.00	5.00

**Table 2:** The values of the declared pleasure of eating various types of dishes, ranging from the most popular ones. Dishes marked in grey are the ones that have been identified by statistical analysis as a common ‘unhealthy food with a strong flavour’ group.

	**N**	**Mean**	**Median**	**SD**	**Minimum**	**Maximum**
Fruit	282	8.63	9.30	1.82	1.00	10.00
Desserts	283	8.24	9.50	2.47	0.00	10.00
Vegetables and salads	282	7.84	8.50	2.26	0.50	10.00
Poultry	282	7.69	8.25	2.29	0.00	10.00
Chocolate products	283	7.61	8.80	2.84	0.00	10.00
Bread	282	7.38	7.80	2.18	0.20	10.00
Pasta	282	7.05	7.60	2.45	0.00	10.00
Egg dishes	283	6.90	7.30	2.61	0.00	10.00
Flour-based dishes	283	6.89	7.10	2.55	0.00	10.00
Soups	283	6.79	7.10	2.58	0.00	10.00
Broth	283	6.72	7.70	3.11	0.00	10.00
Cheeses	281	6.67	7.00	2.72	0.00	10.00
Cured meats	282	6.66	7.20	2.82	0.00	19.00
Fish dishes	283	6.66	7.00	2.67	0.00	10.00
Beef, pork and veal	282	6.54	7.20	3.01	0.00	10.00
Sweets	282	6.24	6.90	3.20	0.00	10.00
Milk products	281	6.03	6.10	2.80	0.00	10.00
Sour products	282	6.00	6.00	2.88	0.00	10.00
Fast food	283	5.71	6.30	3.38	0.00	10.00
Crisps	283	5.58	6.00	3.16	0.00	10.00
Spicy dishes	283	5.47	5.50	3.30	0.00	10.00
Carbonated drinks	282	5.03	5.00	3.11	0.00	10.00
Salty snacks	283	4.84	5.00	2.93	0.00	10.00
Milk soup	282	3.52	2.90	3.14	0.00	10.00
Seafood	283	3.38	2.10	3.42	0.00	10.00

**Table 3:** The effect of predictors such as sex, age, BMI, pack-years, the n-butanol dilution step, odour identification of the group of dishes selected in the factor analysis as Factor One ‘unhealthy food with a strong flavour’; B—Unstandardised regression coefficients; PU—confidence interval; R^2^_c_—multiple determination coefficient; eta^2^-effect size; t—t Statistic; *p*—level of significance.

**Dependent Variables**	**R^2^_c_**	**Predictors**	**B**	**PU**		**t**	**eta^2^**	***p***
Factor One unhealthy food with a strong flavour	0.20	Constant	0.33	-0.51	1.17	0.78	<0.01	0.437
		Sex	0.09	−0.15	0.34	0.76	<0.01	0.448
		Age	−0.04	−0.05	−0.03	−70.43	0.17	<0.001
		BMI	0.03	<0.01	0.06	10.92	0.01	0.056
		Pack-years	0.02	<0.01	0.03	10.77	0.01	0.078
		Olfactory sensitivity	<0.01	<0.01	<0.01	10.06	<0.01	0.288
		Identification test of smell	−0.01	−0.12	0.09	−0.27	<0.01	0.788

**Table 4:** Influence of predictors such as sex, age, BMI, pack-years, n-butanol dilution step, odour identification on the declared pleasure of individual dishes identified in the factor analysis as Factor One ‘unhealthy food with a strong flavour’; B—Unstandardised regression coefficients; PU—confidence interval; R^2^_c_—multiple determination coefficient; eta^2^-effect size; t—t Statistic; *p*—level of significance.

**Dependent Variables**	**R^2^_sk_**	**Predictors**	**B**	**PU**		**t**	**eta^2^**	***p***
Crisps	0.21	Constant	7.78	5.18	10.38	5.90	0.11	<0.001
		Sex	−0.06	−0.82	0.69	−0.16	<0.01	0.871
		Age	−0.12	−0.15	−0.09	−7.45	0.17	<0.001
		BMI	0.07	−0.02	0.17	1.54	0.01	0.125
		Pack-years	0.02	−0.04	0.07	0.64	<0.01	0.520
		Olfactory sensitivity threshold	<0.01	<0.01	<0.01	0.73	<0.01	0.466
		Identification test of smell	−0.10	−0.43	0.23	−0.59	<0.01	0.553
Salty snacks	0.09	Constant	6.12	3.54	8.70	4.67	0.07	<.001
		Sex	−0.27	−10.02	0.48	−0.72	0.00	0.473
		Age	−0.07	−0.10	−0.04	−40.11	0.06	<0.001
		BMI	0.02	−0.07	0.12	0.51	<0.01	0.608
		Pack-years	0.02	−0.03	0.08	0.87	<0.01	0.385
		Olfactory sensitivity threshold	<0.01	<0.01	<0.01	0.80	<0.01	0.422
		Identification test of smell	0.09	−0.24	0.41	0.51	<0.01	0.610
Fast food	0.26	Constant	6.47	3.77	9.17	4.72	0.07	<0.001
		Sex	0.97	0.18	1.75	2.43	0.02	0.016
		Age	−0.14	−0.17	−0.11	−8.22	0.20	<0.001
		BMI	0.09	−0.01	0.19	1.72	0.01	0.086
		Pack-years	0.02	−0.04	0.08	0.64	<0.01	0.522
		Olfactory sensitivity threshold	<0.01	<0.01	<0.01	−0.08	<0.01	0.937
		Identification test of smell	−0.01	−0.35	0.34	−0.03	<0.01	0.976
Sugary carbonated drinks	0.09	Constant	4.26	1.50	7.01	3.04	0.03	0.003
		Sex	1.25	0.45	2.05	3.07	0.03	0.002
		Age	−0.06	−0.09	−0.02	−3.38	0.04	0.001
		BMI	0.01	−0.10	0.11	0.10	<0.01	0.918
		Pack-years	0.05	−0.01	0.11	1.59	0.01	0.113
		Olfactory sensitivity threshold	<0.01	<0.01	<0.01	0.33	<0.01	0.745
		Identification test of smell	0.15	−0.20	0.50	0.85	<0.01	0.398
Sour products	0,04	Constant	3.20	0.57	5.83	2.39	0.02	0.017
		Sex	0.33	−0.44	10.09	0.85	<0.01	0.398
		Age	0.02	−0.01	0.06	1.38	0.01	0.169
		BMI	0.03	−0.07	0.13	0.64	<0.01	0.526
		Pack-years	<0.01	−0.05	0.06	0.11	<0.01	0.910
		Olfactory sensitivity threshold	<0.01	<0.01	<0.01	2.29	0.02	0.023
		Identification test of smell	0.20	−0.14	0.53	1.14	<0.01	0.254

## Data Availability

The datasets used and/or analyzed during the current study are available from the corresponding author on reasonable request.
